# Cementless Total Hip Arthroplasty with Medial Wall Osteotomy for the Sequelae of Septic Arthritis of the Hip

**DOI:** 10.4055/cios.2009.1.1.19

**Published:** 2009-02-06

**Authors:** Myung Chul Yoo, Yoon Je Cho, Kang Il Kim, Kee Hyung Rhyu, Young Soo Chun, Sung Wook Chun, Hoon Oh, Eun Yeol Kim

**Affiliations:** Department of Orthopaedic Surgery, College of Medicine, Kyung Hee University, Seoul, Korea.

**Keywords:** Sequelae of septic arthritis of the hip, Cementless THA, Circumferential medial wall osteotomy

## Abstract

**Background:**

We performed a retrospective study to evaluate the results of acetabular circumferential medial wall osteotomy, a procedure designed to provide secure fixation of a cementless hemispherical acetabular cup for the sequelae of septic arthritis of the hip.

**Methods:**

We assessed 38 total hip arthroplasties (THAs) with circumferential acetabular medial wall osteotomies performed on patients with sequelae of septic arthritis of the hip between 1993 and 2000, who were followed up for ≥ 3 years. The average follow-up period was 8.3 years (range, 3 to 12 years). The indication for this technique was poor acetabular cup coverage of ≤ 70% on preoperative templating. In all cases, cementless hemispherical acetabular cups were fixed to the true acetabulum. Additional procedures included soft tissue release in 16 hips and femoral derotational and shortening osteotomies in 12 hips. We evaluated both clinical and radiological results.

**Results:**

The Harris hip scores improved from 57 points preoperatively to 91 points postoperatively. Radiological analysis revealed no aseptic loosening or radiolucent lines around the acetabular cup. Stable bony fixation of the acetabular cup in the true acetabulum was seen in all cases. Acetabular osteolysis was demonstrated in 12 hips. Revision surgery was performed in 6 hips, but there were no complications related to acetabular circumferential medial wall osteotomy.

**Conclusions:**

Circumferential acetabular medial wall osteotomy can provide appropriate positioning and sufficient coverage of the acetabular cup and thus preserve the medial wall thickness in cementless THA without the need for additional bone grafting for the sequelae of septic arthritis of the hip.

Total hip arthroplasty (THA) for the late sequelae of childhood septic arthritis of the hip is a technically challenging procedure because of underdevelopment and severe deformation of the acetabulum, insufficient bone stock in the superolateral acetabulum causing insufficient coverage of the acetabular cup, and non-anatomical positioning of the hip contributing to accelerated aseptic loosening.^1^,2) The described methods designed to address these problems include implantation of a small acetabular component,^3^-7) medial displacement of the acetabular cup by fracture of the medial acetabular wall in conjunction with a bone graft or cement,^8^-12) and acetabuloplasty using a structural femoral head autograft or allograft.^13^-15) However, bone resorption and aseptic loosening of the acetabular component are still leading causes of poor long-term results with the most preferred technique, which is acetabuloplasty using a bone graft for acetabular deficiency. We attempted to solve these problems and to obtain stable fixation of the cementless acetabular cup without using bone grafts, while still preserving the thickness of the medial acetabular wall. Therefore, we developed a new technique: circumferential osteotomy of the medial acetabular wall. The purpose of this study was to report the clinical and radiological results of this procedure performed on patients with steep and shallow acetabula as sequelae of septic arthritis of the hip.

## METHODS

### Materials

This study involved 38 patients (38 hips) who were available for a minimum 3-year follow-up after THA with circumferential osteotomy of the medial acetabular wall. Patients underwent this procedure between October 1993 and March 2000 for the treatment of sequelae related to childhood septic arthritis of the hip. There were 13 men and 25 women, with a mean age of 44 years (range, 23 to 66 years). The mean follow-up period was 8.3 years (range, 3 to 12 years).

**Fig. 1:**
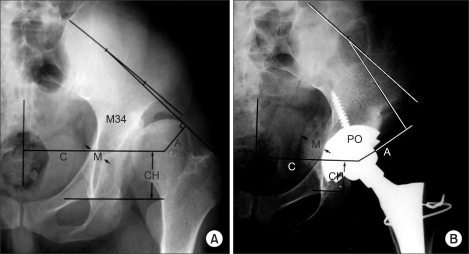
Anteroposterior roentgenogram measurements in preoperative (A) and postoperative (B) diagrams of the pelvis. A: Abductor moment arm, M: Thickness of the medial wall of the acetabulum, C: Center body moment arm, CH: Center of hip.

In all patients, the acetabular cup was placed in the true acetabulum. In 16 hips, soft tissue release was performed to reduce the dislocated hip and to ensure proper joint motion after surgery. In 12 patients, distal femoral shortening and derotational osteotomy were performed concomitantly. For the purpose of prevention of the sciatic nerve injury during the trial reduction, correction of anteversion to prevent postoperative dislocation. Additional structural bone grafting was carried out in 8 patients who had ≤ 70% coverage of the acetabular component even after circumferential osteotomy. Cementless porous coated acetabular cups were used in all hips: Harris-Galante I (Zimmer, Warsaw, Indiana, USA) in 10 hips and Harris-Galante II in 28 hips. The results were evaluated clinically using the Harris hip score (HHS) and radiologically using roentgenograms at every follow-up. All anteroposterior and translateral views were assessed for acetabular cup fixation, osteolysis, loosening, radiolucent lines, implant migration, hip center, ratio of body weight lever arm to abductor lever arm, thickness of the medial acetabular wall, and bony union and remodeling of the osteotomy site (Fig. 1). Bony union was defined as the absence of a lucent line at the osteotomy site. Bone remodeling was assessed based on the presence of bone trabecula and changes in the thickness of the medial wall.

### Indications

Indications for surgery were defined as an abnormally steep and shallow acetabulum, ≤ 70% acetabular coverage due to insufficient bone stock in the superolateral acetabular wall, relatively clear pepripheral rim of the acetabular fossa, and ≥10 mm thickness of the medial acetabular wall on radiography.

### Surgical Technique

**Fig. 2:**
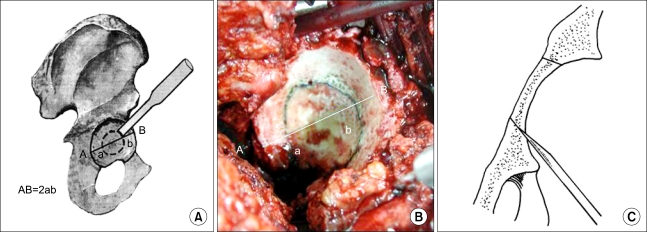
The line of the circumferential medial wall osteotomy. Schematic view (A) and intraoperative view (B). Osteotomy direction of the procedure (C).

Circumferential acetabular medial wall osteotomy, which was developed by the first author of this paper, was designed to provide compressive fixation of the cementless acetabular component in the dysplastic acetabulum without compromising the thickness of the medial wall and causing damage to the medial acetabular wall. Such damage usually results from excessive expansion during reaming for improving coverage of the cementless acetabular cup. Circumferential acetabular medial wall osteotomy allows medial displacement of the center of the hip for biomechanical balance. In all cases, surgery was performed via a posterolateral approach. Removal of inflammatory scar tissue and contracted pseudoarthrosis tissue of the hip was followed by soft tissue release around the acetabulum, which allowed for identification of the sharp and steep true acetabulum. The acetabular cartilage was resected with a reamer, and a narrow hole was drilled into the medial wall of the center of the acetabulum in order to measure the thickness. A circumferential line was then drawn in the middle between the center of the true acetabulum and the acetabular margin. An osteotomy was performed along this equidistant line (Figs. 2A, B). During this procedure, the osteotome was inclined 20° towards the centre of the acetabulum to form the fragment into a wedge shape (Fig. 2C).

**Fig. 3:**
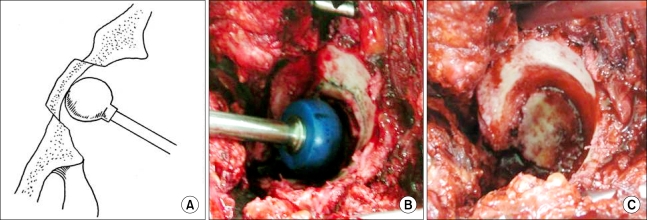
Pushing the osteotomized medial wall with a cup pusher after circumferential medial wall osteotomy. Schematic view (A) and intraoperative view (B). The medial wall was displaced medially, and thus the shallow acetabulum became deeper (C).

**Fig. 4:**
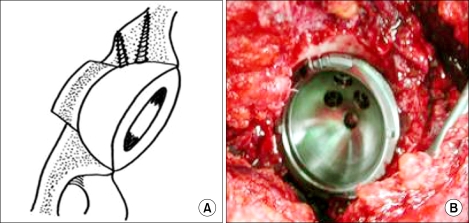
Completely seated porous coated hemispherical acetabular cup after reaming of the acetabular fossa. Schematic view (A) and intraoperative view (B).

This was intended to prevent excessive medial displacement and to obtain stability of the osteotomized fragment without additional external support. The frag-ment was medially impacted by a cup pusher by 2/3 the thickness of the acetabular wall (Figs. 3A, B). As the osteotomized fragment was of a conical shape, it could be stably fixed by the adjacent acetabulum when pushed into the pelvis (Fig. 3C). And then reaming was carried out very carefully starting with the smallest reamer and increased the size of reamer gradually while taking care to retain as much of the subchondral bone as possible. Finally, compressive fixation of a cementless hemispherical cup, 2 mm larger than the reamed acetabular fossa, was performed (Fig. 4).

Postoperative treatments were almost the same as those of the established THA. Quadriceps femoris muscle strengthening exercises were started 2 to 3 days after the surgery. Partial weight-bearing using crutches began 5 to 7 days after surgery, and full weight-bearing was allowed at 3 to 4 months. At every follow-up visit, bony union of the medial acetabular wall and remodeling were assessed on pelvis inlet views, in addition to anteroposterior and translateral radiographs and CT in some cases.

## RESULTS

**Fig. 5:**
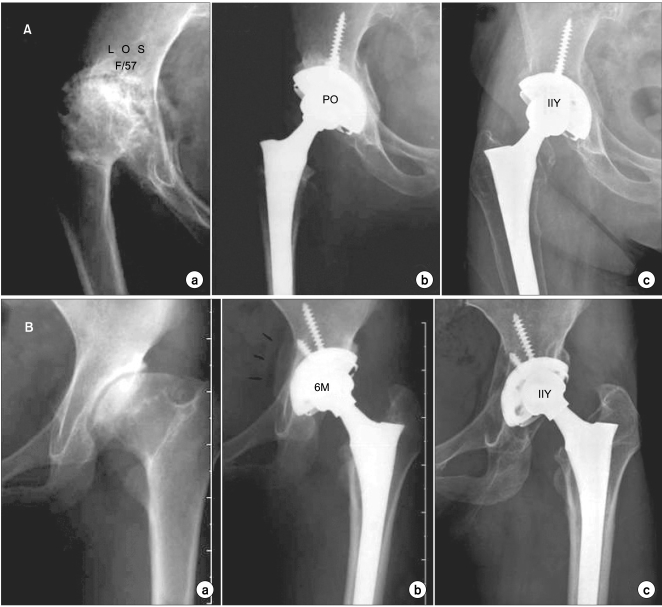
(A) Preoperative (a) and postoperative (b) radiographs in a 57-year-old woman's hip in which a medial wall osteotomy was used in THA to obtain adequate coverage of the acetabular component and a natural center of rotation. (c) Anteroposterior radiograph, taken 11 years postoperatively, showing thick bone stock of the well remodeled medial acetabular wall. (B) Preoperative (a) and 6-month postoperative (b) radiographs in a 21-year-old woman's hip in which a medial wall osteotomy was used in THA to obtain adequate coverage of the acetabular component and a natural center of rotation. (c) Anteroposterior radiograph, taken 11 years postoperatively, showing thick bone stock of the well remodeled medial acetabular wall.

The average Harris hip score improved from 57 points (range, 35 to 84 points) preoperatively to 91 points (range, 62 to 100 points) at the latest follow-up. In all hips, stable bony fixation of the acetabular component at the level of the true acetabulum was identified on radiographs (Fig. 5). The height of the center of the hip, defined as the distance from the interteardrop line to the center of the femoral head, decreased by 14 mm on average from a preoperative mean of 34 mm (range, 22 to 68 mm) to a postoperative mean of 20 mm (range, 11 to 30 mm). In other words, the hip center was brought back down to its anatomical position in almost all hips. Medialization of the acetabular cup resulted in a increase in the relative length of the abductor moment arm. Accordingly, the ratio of the body weight lever arm to the abductor lever arm changed from a preoperative mean of 3.0:1 (range, 1.9:1 to 3.9:1) to a postoperative mean of 2.2:1 (range, 1.4:1 to 3.2:1) (Table 1), a statistically significant change (*p* = 0.003).

During the follow-up period, bony union of the osteotomized medial acetabular wall was observed at an average of 4 months (range, 3 to 5 months) postoperatively, and bone remodeling occurred at a mean of 7 months (range, 6 to 12 months) postoperatively.

At the latest follow-up, osteolysis was noted in 12 hips (31.5%). It was observed in DeLee and Charnley zone I in 6 hips, zone II in 6 hips, and zone III in 3 hips. There was no elevated incidence of osteolysis in zone II where the osteotomy had been performed. Nine hips had osteolysis in one zone, and three hips had osteolysis in two zones. No case exhibited loosening of the acetabular cup or appearance of a radiolucent line. The mean annual polyethylene wear rate was 0.18 mm as measured by the Livermore11) technique. Six hips underwent revision. In two hips with excessive polyethylene wear, the polyethylene liner was exchanged with bone grafting into the osteolytic lesion of acetabular sides retaining the acetabular cup. In two other hips that were revised for dissociation of the polyethylene liner, one required exchange of the polyethylene liner (but not the acetabular cup) along with acetabular bone grafting; acetabular cup revision surgery was unavoidable in the other hip due to damage to the cup. Femoral stem revision was necessitated by severe osteolysis around the stem in one hip. Deep infection made it mandatory to replace the acetabular cup and the femoral stem in one hip. The acetabular cup had to be revised in two hips. Until the final follow-up, not a single hip required revision arthroplasty due to fixation failure of the acetabular cup.

## DISCUSSION

THA is regarded as the most effective treatment for the late sequelae of childhood septic arthritis of the hip. However, it is a technically challenging procedure.^1^,16) The application of this technique is hampered by anatomical changes, such as severe dysplasia, deformity of the acetabulum, and formation of a false acetabulum resulting from dislocation or subluxation of the femoral head. A variety of procedures have been introduced in the interest of reconstructing the acetabulum, including the use of cemented cups or cementless acetabular cups and femoral head bone grafting or medialization of the acetabular cup. Charnley and Feagin17) recommended the use of small cemented prostheses on the grounds that loosening and laxity of the joint can be reduced when the lack of coverage for the superolateral portion of the cup is < 5 mm. Harris et al.^13^,18) tested a procedure in which the excised femoral head was fixed with bolts to the superolateral acetabular wall for the augmentation of the deficient acetabulum, but found that resorption of the graft resulted in a drastic increase in loosening of the acetabular cup during long-term follow-up.^18^-20)

Spangehl et al.21) used cementless acetabular cups and bone grafts for acetabular reconstruction in 44 dysplastic hips and noted no resorption of the graft or loosening during the mean 7.5-year follow-up period, unlike that seen with other cemented bone graft procedures. Four revisions were required. In the current study, the authors found no loosening of the acetabular cup during the average 8.3-year follow-up. The causes of the six revisions included polyethylene wear, dissociation of the polyethylene implant (a common defect of the Harris-Galante acetabular cup), severe osteolysis around the femoral stem, and deep infection. Exchange of the acetabular cup was required in two cases where either dissociation of the polyethylene liner caused severe damage to the metal acetabular cup or infection occurred. Both cases showed no evidence of acetabular cup loo-sening. Until the final follow-up, compressive fixation of the acetabular cup was so successful that loosening was not observed. The osteotomized fragment in the middle of the acetabulum was not completely displaced, but was partially abutting against the adjacent acetabulum, and the medial periosteum was left intact. Accordingly, blood flow was normal, and bony union was obtained. Stable bony ingrowth was assumed in the absence of a radiolucent line in zone II.

Johnston et al.22) and Russotti and Harris23) concluded in their experimental study on the center of the hip that the non-anatomical position of the acetabular cup is mainly responsible for loosening of the acetabulum. Johnston et al.22) put emphasis on the anatomical position of the acetabular cup on the grounds that when it is located 20 mm lateral, 20 mm proximal, and 10 mm posterior to the anatomical site, 116% more abductor muscle strength is required in normal walking. Among the many authors who tried medial displacement of the acetabular cup,^6^,^22^-24) Dunn and Hess8) suggested that placing the acetabular component closer to the true acetabulum prevents loosening and Trendelenburg gait. In their study, a controlled fracture of the iliac wall or the medial acetabular wall was created to medialize the acetabular component for increased acetabular coverage. The problems noted in the study included unstable fixation of the acetabular cup resulting from loss of subchondral bone after excessive reaming of the medial acetabular wall and fracture caused by weakness of the medial wall during follow-up.^4^,25)

Hartofilakidis et al.9) reported successful results using medialization of the acetabular cup in conjunction with creation of a controlled radiating fracture of the true acetabulum, bone grafting, and bone cement. The success rate was 92.4% at 5-year follow-up and 88% at 10-year follow-up. The investigators concluded that the improvement was attributable to the lengthening of the abductor lever arm. Dorr et al.26) reported that medialization of the cementless acetabular cup led to no revision arthroplasty during a mean 7-year follow-up period in their study. Although bone grafting can be avoided with such a technique, excessive reaming may cause the medial wall to be prone to fracture and the acetabular component to be prone to failure through mechanisms such as medial migration. In addition, the thin medial wall poses a challenge in revision surgery for replacing the acetabular cup. With circumferential osteotomy, we were able to avoid the problems that can occur when the medial acetabular wall is excessively reamed for fixation of the cementless cup. Our technique enabled the acetabular cup to be fixed to the anatomical position of the acetabulum while maintaining the thickness of the medial wall without damage. Therefore, risk factors for fracture and medial migration of the acetabular cup were avoided, and the ratio of the body weight lever arm to the abductor lever arm was reduced.

In the study of Torisu et al.27) investigating THA without acetabular bone grafting in patients with degenerative coxarthritis resulting from dysplastic acetabulum, bony union was enhanced through preservation of the subchondral bone and expansion of the host-graft interface. Hence, factors that could lead to acetabular cup failure, such as fracture and medial migration, were eliminated. These investigators also reported that the thickness of the medial wall should be ≥ 10 mm in order to achieve stable fixation of the acetabular component by bone remodeling without cement. In the index study, the indication for our technique with regard to the thickness of the medial wall was ≥ 10 mm. The average thickness was 13 mm preoperatively and 11 mm postoperatively, which suggested the presence of minor changes in the acetabular wall thickness contributing to stable fixation of the acetabular component in a safe way (Table 1).

The condition of the medial wall plays a role in the level of postoperative medial displacement of osteotomized fragments, so exact quantification is difficult. That is why the surgeon's skill should not be taken lightly. The osseous interface between the medialized fragment and the rest of the acetabulum should be properly maintained to achieve satisfactory bony union after surgery. In our procedure, the intact subchondral bone allowed for the use of a press-fit acetabular component. Furthermore, the preservation of the medial acetabular wall prevented medial migration of the cup, and the maintained thickness of the medial wall allowed for sufficient bone remodeling.

Cementless THA with circumferential osteotomy of the medial acetabular wall in patients with late sequelae of childhood septic arthritis of the hip provided enough coverage of the acetabular cup without the need for bone grafting. Stable fixation of the cup was achieved, and the medial wall thickness was preserved. At the latest follow-up, bony ingrowth into the acetabular cup, biological fixation, and remodeling of the osteotomized medial wall were identified on radiographs. Therefore, we believe our technique could be beneficial in securing stable fixation of the cementless acetabular component, implantation in the true acetabulum, and sufficient coverage of the acetabular cup, without impact on the thickness of the medial acetabular wall. Furthermore, we suggest this technique as a valuable method for biomechanically improving the hip joint by medializing the hip center and lengthening the abductor lever arm.

## Figures and Tables

**Table 1:**
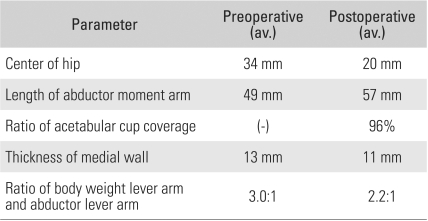
Radiologic Parameters on Follow-up Evaluation
